# Book Reviews

**Published:** 1899-08

**Authors:** 


### *BOOK REVIEWS, PAMPHLETS, EXCHANGES.*

### *BOOK REVIEWS.*

**[Contributions solicited for review.]**

TEXT-BOOK OF OPHTHALMOLOGY. By Dr. Ernest Fuchs, Professor of Ophthalmology in University of Vienna. Translated from the seventh enlarged edition by A. Duane, M.D., of New York. Second American edition. Published by D. Appleton & Co., New York.

This second American edition has just been issued by the Appletons. This work of Professor Fuchs's has for years enjoyed an unusual popularity which has been well deserved. Dr. Duane is also to be congratulated upon his work as translator. We have for some time held this to be one of the best text-books on ophthalmology to which it has been our pleasure to refer, and as a ready reference book it has many things to commend itself. We like the arrangement and the clearness with which each subject is presented. The corollaries in small type, usually found after the subject has been treated, are gems of practical information. The illustrations are all good, and with the additions made by Dr. Duane upon the subject of heterophoria and the use of homatropin the book is thoroughly up to date.

The sheet-anchor treatment, according to the writer, is the making and keeping of the nasal and nasopharyngeal mucous membrane in an antiseptic condition.

He then gives the solution which he uses to accomplish this condition, but *mirabile dictu,* listen to the technique: "This I thoroughly use in both nostrils; first by means of a hand-ball atomizer; after which, with a curved aluminum applicator or a Harrison Allen's nasal cotton-carrier, I very carefully swab the whole nasopharynx." Think of "swabbing thoroughly" the whole nasopharynx of a patient suffering with hay fever whose respiratory mucous membrane is sensitive to a degree! Think of being able to swab every little corner as the writer suggests! It reminds us of putting salt on the bird's tail to catch the bird. Listen again, and they are words in italics: "*I scrub most carefully and gently every portion of the mucous membrane . . . . . .*" Think of scrubbing the turbinates in a fully developed case where the nasal cavity is as tight as a drum and watery mucus running out by the ounce! It is hard enough after the thorough use of cocain to barely touch the nasal mucous membrane, much less "scrub" it. Listen further: "I then as carefully dry the membrane with clean cotton." Ye gods, think of drying the mucous membrane of a fully developed hay fever case. Surely the writer must have intended the chapter on treatment for novices or those who never saw a case. And yet, after all, in the language of our Georgia statesman, "where are we at"?

---

THE MEDICAL COMPLICATIONS, ACCIDENTS AND SEQUELÆ OF TYPHOID OR ENTERIC FEVER. By H. A. Hare, M.D., B.Sc., Professor of Therapeutics in the Jefferson Medical College, Philadelphia. With a special chapter on the Mental Disturbances Following Typhoid Fever. By F. X. Dercum, M.D., Clinical Professor of the Nervous System in Jefferson Medical College. Published by Lee Brothers & Co. Philadelphia and New York.

Like all the writings which have come from the pen of Dr. Hare, this brochure is no exception in being fully up to the standard. To one whose work and practice embraces a large number of typhoid patients this little book will be of exeeptional value as it is filled with much scientific and practical matter. All will agree with Dr. Hare, who says in his preface: "At the present time there are few diseases so widespread as typhoid fever, and the literature concerning it is very great. Systems of medicine and text-books innumerable deal with its ordinary manifestations, and touch, necessarily but briefly, upon its accidents, its complications, and its sequelæ." Chapter I deals with "General Conditions," embracing its topography and its widespread prevalence in the various countries of the world. Chapter II deals with "Varieties of Onset," giving the temperature charts showing its great and manifold density. Chapter III deals with the complications which arise when the disease is well developed. Chapter IV

treats of the complications during convalescence. Chapter V takes in those conditions which resemble typhoid. Chapters VI and VII deal with duration and immunity to second attacks and the mental complications.

---

HAY FEVER AND ITS SUCCESSFUL TREATMENT. By W. C. Hollapeter, A.M., M.D., Professor of Pediatrics in the Medico-Chirurgical College, Philadelphia. Second edition. Published by P. Blakiston's Son & Co. Philadelphia.

The second edition of this brochure is before us. Our views and criticisms have not changed since the first review last year. Why there should ever have been a call for a second edition will ever remain a mystery to us. The first part of the book, which deals with the history of hay fever, its causes, symptoms, diagnosis, prognosis, is of value to the rhinologist because it treats the subject possibly a little more exhaustively than can be found in the majority of text-books upon nasal diseases, but the chapter on treatment and many of the statements therein contained savor of the veriest rot. How any man could ever imagine that such statements and line of treatment as are contained in this book could ever be seriously considered by any one who had ever had any practical experience whatever in the treatment of hay fever, is beyond our conception. Listen at such a statement as this found on page 101: "But we can unhesitatingly say that a majority of cases are curable, and that positive relief, without change of residence or inconvenience, can be afforded during the period of occurrence if treatment is directed along the lines laid down in the following chapter." That some cases are curable and many benefited is known to every rhinologist of any experience, but such bold statements as the above savor of charlatanry.

---

AN EXPERIMENTAL RESEARCH INTO SURGICAL SHOCK. An Essay awarded the Cartwright Prize for 1897. By Geo. W. Crile, A.M., M.D., Ph.D., Professor of the Principles of Surgery and Applied Anatomy in the Cleveland College of Physicians and Surgeons. Published by the J. B. Lippincott Co. Philadelphia.

This brochure, as its name indicates, is the putting in printed form the results obtained in an experimental research conducted on animals for the purpose of enlarging our knowledge on the question of "shock." In all one hundred and forty-eight animals were subjected to experiment. The work is, of course, largely scientific. Charts are given showing the blood pressure and respiratory changes which occurred when the animal was subjected to an operation in various parts of the body. If we can gather anything as to the pathology of shock by noting such respiratory and blood pressure changes, then the author's work is certainly of value, but we are afraid that as yet it is a subject *incognito.*

---

THE NEWER REMEDIES, INCLUDING THEIR SYNONYMS, SOURCES, METHODS OF PREPARATION, TESTS, SOLUBILITIES, INCOMPATIBLES, MEDICINAL PROPERTIES AND DOSES AS FAR AS KNOWN, TOGETHER WITH SECTIONS ON ORGANO-THERAPEUTIC AGENTS AND INDIFFERENT COMPOUNDS OF IRON. By Virgil Coblentz, A.M., Pharm. M., Ph.D. Third edition. Published by P. Blakiston's Son & Co. Philadelphia. Price $1.00.

This is an exceedingly valuable little book, especially for pharmacists and physicians whose work is more especially in the realm of therapeutics. The remedies are classified alphabetically, and their chemical constitution clearly given. A short history is sometimes given. The remedies are all the newest up-to-date.

